# Intervention and experiment

**DOI:** 10.1007/s13194-025-00647-3

**Published:** 2025-03-13

**Authors:** Irina Mikhalevich

**Affiliations:** North Carolina State University, Raleigh, NC 27695 United States

**Keywords:** Experiment, Intervention, Observation, Natural experiment, Causation, Evidence, Control

## Abstract

The received view of scientific experimentation holds that science is characterized by experiment and experiment is characterized by active intervention on the system of interest. Although versions of this view are widely held, they have seldom been explicitly defended. The present essay reconstructs and defuses two arguments in defense of the received view: first, that intervention is necessary for uncovering causal structures, and second, that intervention conduces to better evidence. By examining a range of non-interventionist studies from across the sciences, I conclude that interventionist experiments are not, *ceteris paribus,* epistemically superior to non-interventionist studies and that the latter may thus be classified as experiment proper. My analysis explains why intervention remains valuable while at the same time elevating the status of some non-interventionist studies to that of experiment proper*.*

## Introduction

The received view of scientific activity holds that science is characterized by experiment and that experiment is characterized by active intervention on the system of interest (Bacon, 1878; Giere, 2009; Franklin, 1990; Hacking, 1983; Tiles, 1993; Peschard, 2012; Merchant, 2013; Zwier, 2013). Although this view appears to be widely held, it has seldom been explicitly defended. Moreover, many scientific studies do not involve intervention, and so represent a challenge to the received view. These include natural experiments in the historical sciences (e.g., paleontology, anthropology) and fields where ethical constraints prevent intervention (e.g., economics, political science, epidemiology); observational studies (e.g., in astronomy and ethology); and model-based and simulation experiments (e.g., in climate studies and experimental biology). None of these studies involve intervention on the target system: Models and simulations intervene only on proxy systems, such as model organisms or computational climate models. Observational studies involve the manipulation only of measurement instruments, such as telescopes and audio recorders, but not of the objects of study, such as planetary orbits or baboon social hierarchies. And natural experiments involve only what might be called “pseudo-interventions,” or instances where nature or society effectively provides the conditions that an experimenter would have brought about had she had the ability, or where the scientist deploys statistical analyses to retrospectively segregate populations into treatment and control groups, thus effectively controlling for confounds. Yet these non-interventionist studies can successfully explain phenomena, promote understanding, and lead to novel discoveries. In other words, they appear to do the epistemic work commonly attributed to experiment.

Non-interventionist studies have received extensive philosophical attention (e.g., Cleland, 2002; Giere, 2009; Morrison, 2009; Parke, 2014; Parker, 2009; Sekhon & Titiunik, 2012; Waters, 2007; Weber, 2014; Winsberg, 2003; Winsberg, 2010), but most discussions have focused on a single kind of non-interventionist study and few have explicitly challenged or defended the received view in their analyses.^1^ Even those who argue that certain models, simulations, or natural experiments may be epistemically on a par with experiment proper often assume that experiment requires intervention and that experimentation is all-things-considered superior to non-experimental studies. However, as I will suggest, not only is the received view unjustified in excluding noninterventionist studies from the class of experiments proper, but such exclusion is potentially harmful to scientific research programs and individual researchers. In particular, the label of ‘experimental science’ affords fields a degree of prestige from which critical resources, such as opportunities for funding and institutional support, tend to flow. If valuable resources are tied to perceptions of experimentality, and if experimentality is understood in interventionist terms, then practitioners of fields in which intervention is either impossible or methodologically inappropriate might feel incentivized to adopt suboptimal interventionist research strategies.

This essay with defend the heterodox view that non-interventionist studies may be epistemically on a par with interventionist experiment and can therefore be classified as experiment proper. After presenting the received view (§2) I will reconstruct and defuse two arguments for the epistemic superiority of intervention: first, that intervention uniquely permits causal discovery (§3); and second, that it delivers better evidence (§4). Then, in §5, I suggest that while intervention offers unique advantages, these advantages are of practical rather than epistemic value. Finally, §6 sketches a revised and more inclusive account of experimentation on which what sets experiment apart is not intervention but control, which may be attained through what I call “deliberate positioning.” My analysis thus explains the unique value of intervention while plausibly elevating some non-interventionist studies to the status of experiments proper*.*

## Intervention and the received view of experiment

What I have been referring to as the received view consists of the following three interrelated theses:

- Experimentation is central to science
- Interventionist scientific studies are (ceteris paribus) epistemically superior to non-interventionist studies *(epistemic superiority thesis)*
- Experiment requires intervention on the target system *(experiment as intervention thesis)*; non-interventionist studies are not experiments proper.^2^

In what follows, I will set the question of whether experimentation is central to science aside and focus on theses b (in §3 and §4) and c (in §5 and §6). Toward this end, I will adopt Peschard’s (2012) definition of intervention, on which:

- **Intervention** = “deliberate and targeted perturbation of any physical system (organism, group, molecular compound, …, etc.), conducted in any possible location (laboratory, field…) that is intended to observe and measure changes in that system for the purposes of attaining new knowledge about that system” (Peschard, 2012).

Intervention in this sense is a type of physical manipulation that is intentional, targeted, and carried out on the system of interest in order to further the epistemic goal of an agent. Alternative views of intervention, such as those that are neutral with respect to the ontology of the target system or the involvement of an intentional agent, may issue in different conclusions about the value of intervention or its role in experiment.^3^ However, this ‘thick’ account of intervention captures the sense in which the term is deployed in much of the literature on experimentation and in common discourse. For example, simulations and models are commonly considered to be non-interventionist because the deliberate and targeted perturbations are not made on the target system or a representative sample of the target system.^4^ Similarly, while natural experiments involve targeted physical perturbations of the system of interest, these perturbations are produced by nature or by society rather than by a human agent. With this account of intervention in mind, let us now turn to two arguments for the epistemic superiority thesis, beginning with the claim that intervention is necessary for causal discovery.

## Intervention and causation

Causation, in one form or another, is at the heart of many philosophical models of scientific explanation (Woodward & Ross, 2021). For example, according to the influential “new mechanist” accounts of explanation in the neurobiological and cognitive sciences, scientific explanation involves the identification of the “causal structures that produce, underlie, or maintain the phenomenon of interest” (Craver et al., 2015). At the same time, so-called interventionist theories of causation seek to define causal relationships in terms of actual or hypothetical interventions in the system of interest (Baumgartner, 2009). *If* causal relationships are central to scientific explanation and *if* uncovering causal relationships requires intervention, then intervention would seem to be necessary for scientific explanation, lending support to the epistemic superiority thesis.^5^

While I will not mount a challenge to the notion that causal relationships are central to scientific explanation, I will note that significant swaths of scientific activity, including measurement studies and preparatory experiments, aim to uncover non-causal (e.g., mereological) relationships and that interventionist techniques are often inappropriate within these studies. For example, Kästner (2017) argues that even within paradigmatically mechanistic sciences, such as neuroscience, strategies such as cell staining, MRI studies, and microscopy, which aim to identify constitutive elements of the system may play a significant and often overlooked role. These studies are often a prerequisite for causal interventions, as we must first know *what* to intervene upon and where to target the intervention. Kästner refers to such studies as *mere interactions*: where interventions bring about changes to the system, mere interactions bring elements of the system into view, often in preparation for causal-interventionist experiments. These techniques interact with systems without augmenting their functional character, provided that the studies do not alter what Currie and Levy (2019) refer to as the “focal properties” of the systems by mistake. As such, they are more akin to instrument-mediated observation than to intervention in the thick sense. Indeed, *causal* interventions could alter the functional parameters of the system before they could be measured, and are thus not only unhelpful, but potentially destructive.

More importantly, uncovering causal relationships may not require intervention. For instance, imaging studies use radioactive markers, or tracers, to aid in the visualisation of not only constitutive relationships such as neuron populations, but in the understanding of causal biological processes such as neural cascades (Ross, 2021). Natural experiments, too, may be capable of identifying causal relationships without intervention. Initially coined by the epidemiologist, John Snow (1813–1858), “natural experiment” refers to fortunate setups in nature or society where the relevant variables have been effectively ‘controlled’ by nature or society rather than by the active intervention of a researcher. They are common in historical sciences (e.g., paleontology, geology), where intervention is often impossible, and in fields such as epidemiology, economics, and political science, where intervention may be unethical or impractical. Paleontologists interested in explaining extinction-level events, epidemiologists seeking to understand the drivers of the COVID-19 pandemic, and political scientists studying the effects of redistricting on voting patterns (Sekhon & Titiunik, 2012), cannot intervene to bring about the conditions they wish to study and must instead search for instances in nature or society that replicate the conditions of interest.

To see how natural experiments may lead to causal discovery consider the famous example of Snow’s discovery that water delivery systems were responsible for the great cholera epidemic in nineteenth century London (Snow, 1856). The bulk of Snow’s efforts consisted in reasoning and measurement rather than intervention: for instance, when Snow plotted the affected households on a map of the city, what emerged were patterns of infection that precisely tracked differences in water-delivery systems. In one investigation, he traced the pattern back to a common well; in another, to the company that supplied water which had been contaminated upstream. In effect, society had inadvertently segregated Londoners into what we would today call “treatment” and “control” groups in both investigations: those who received contaminated water and those who did not.^6^ Moreover, because the source of water-delivery for a given house was effectively random insofar as it did not track such factors as class membership, the natural experiment had effectively controlled for confounding variables in “assigning” group membership.^7^ Based on these maps, Snow was able to conclude that cholera was caused by contact with contaminated water. Notice that both the segregation into treatment and control groups and the control for confounds was delivered not by active intervention, but by chance set-ups that became apparent only with Snow’s skilled analysis. Snow’s conclusion that the cholera outbreaks were caused by contaminated water is thus one example of how natural experiments might generate causal discovery.

One might object that Snow did not uncover the *cause* of the cholera epidemic, but merely a correlation between water delivery systems and the incidence of cholera, and that all natural experiments, insofar as they rest on similar inferential patterns, are limited to merely correlation rather than causal discovery. To establish causation between (e.g.) water delivery and the incidence of cholera, on this objection, we must not only establish that water delivery systems co-vary with incidences of cholera, but also show *how* water transmits cholera. That is, we need a mechanistic explanation (viz. that the infectious and transmissible bacterium, *vibrio cholerae*, causes the disease) and because natural experiments are unable to offer mechanistic explanation, they are also incapable of causal discovery.

This objection, however, risks circularity by presupposing that all causal explanations must be mechanistic in nature. While a *complete* causal explanation of large-scale events, such as the spread of diseases in a population, may require an understanding of the mechanisms of transmission, a mechanistic analysis is not always necessary and may not be sufficient. For instance, understanding, explaining, and predicting the spread of diseases may require knowledge of patterns of human movement and the infrastructure facilitating them (e.g., airports, railroads, etc.), social customs and practices (e.g., hygiene practices, level of public trust in healthcare authority), political institutions, and more. All of these contribute causally to the spread of diseases, yet none can be rendered in purely mechanistic terms. Second, and relatedly, the objection proves too much, as it could just as well be levelled against interventionist experiments. Consider the following counterfactual: if Snow had personally manipulated the water-delivery systems to deliver cholera-tainted water to a random sample of the population to test the water vector hypothesis for cholera, his results would have been no different. If we are willing to grant that Snow in the counterfactual (morally prohibited) scenario was able to identify the cause of cholera, then we should be willing to do the same for Snow’s natural experiment. What both scenarios have in common, and what secures the causal inference, is that the water-delivery system could be *isolated* as the independent variable. The further question of how water transmits the disease, or, for that matter, what induces cholera-symptoms in a human being, is a separate part of a larger causal story.

Natural experiments may be capable of not only identifying the causes of stochastic events, such as the spread of cholera in a single population or the cause of a single extinction-level event, but also law-like generalizations. Although space prohibits a full exploration of this possibility, one example comes from convergent evolution, or the independent emergence of similar forms and functions from separate origins. Establishing that the same trait emerged independently in several different lineages over the history of life on Earth—e.g., powered flight in birds, insects, and mammals—may permit us to make law-like generalizations about the evolvability of these traits given specific selective pressures and developmental constraints (Currie 2013; Powell & Mariscal, 2015; Powell, 2020). To take one admittedly controversial example, the convergent evolution of cognitive forms among clever vertebrates and at least two invertebrate lineages (coleoid mollusks such as octopuses and squid, and arthropods such as insects and arachnids) may have implications for the evolvability of mind on any life-sustaining world (Godfrey-Smith, 2016; Powell, 2020).

One might object that natural experiments indeed involve intervention, but that it is nature rather than the scientist that performs this intervention. Morgan (2013), for instance, takes this view when she argues that natural experiments can “isolate a process of interest” despite such isolation requiring both intervention and control. If we take this suggestion seriously, however, then we must either attribute agency to nature or revise the meaning of intervention to remove reference to agency, such as the requirements that perturbations should be both *deliberate* and *intended* to induce understanding. The former option appears to implausibly characterize unfolding events—events that lack not only intentionality, but even basic goal-directedness—as agentic.^8^ The latter option, however, reduces intervention to mere causal influence.^9^

Although nature may not intervene, it does produce chance setups that may be exploited by researchers to generate causal knowledge, effectively isolating the target phenomena, and thus controlling for confounds. Morgan (2013) draws a useful distinction between natural experiments in which nature or society effectively control for confounds and cases where confounding causal factors must be controlled *post-facto* through statistical retrofitting or reconstruction by the researcher.^10^ Her distinction suggests that control may be achieved in at least three ways: control by means of human physical intervention; “control” by nature or society (i.e., by chance); and control through human analysis. Snow’s investigations involved the second and perhaps third kinds of control, suggesting that intervention is one strategy among several for effecting control over target phenomena. We return to the subject of control and its relationship to intervention and experimentality in §§4–6.

Finally, investigations in the historical and biological sciences (e.g., geology, archaeology, evolutionary biology, astronomy, palaeontology) may follow patterns of causal inference that are well tailored to their subject matter but that differ from those of traditional interventionist experiment.^11^ For example, Cleland (2002) argues that while experimental sciences seek to predict the effects of causes in order to test regularities in nature, historical sciences seek to explain known phenomena (effects) in terms of long-past causes, in order to explain these singular phenomena. Historical sciences search for what she calls the “smoking gun,” or trace evidence of a past cause that “unambiguously discriminates one hypothesis from among a set of currently available hypotheses as providing ‘the best explanation’ of the traces thus far observed” where the best explanation is the one that unifies the most phenomena under a single causal framework—a scientific virtue that Cleland refers to as “causal unity” (483). If she is right, then even if some causal inferences are less likely to accrue to historical non-interventionist studies, others would remain available. Focusing on just one kind of causal-inferential structure therefore may give the false impression that intervention is the sole means of causal discovery. If different kinds of causal inference (e.g., reasoning from effects to causes versus causes to effects) call for different explanatory strategies, then it would be an error to prioritize one kind of strategy, such as those used in interventionist sciences, over another. Indeed, any view that relegates historical and observational sciences to second class epistemic status must account for the fact that famous instances of revolutionary science, such as Darwin’s theory of evolution by means of natural selection, which he developed after his famous voyage to the Galapagos Islands, were the products not of experimental tinkering but of careful structured observation in the context of a natural experiment. Moreover, since many of the interventionist studies in modern evolutionary biology utilize model organisms, such as bacterial cultures or populations of fruit flies, whose rapid generational turnover permits researchers to observe speciation events in real time, one might fairly conclude that evolutionary biology draws heavily on experimental means that do not fit the description of “intervention” in Peschard’s sense.

Let us take stock. This section has considered the claim that intervention is epistemically superior to non-interventionist techniques because intervention is necessary for causal discovery. I suggested that (1) non-causal relationships are also valuable and that intervention may be a suboptimal strategy for identifying these; and (ii) that non-interventionist techniques can identify causes in the historical, social, and biological sciences and more broadly. Thus, it seems that the received view of experiment cannot advert to the unique ability of intervention to identify causal relationships.

## Intervention and evidence

Suppose we grant that not all explanation in science is causal and that non-interventionist methods can in principle lead to causal discovery. One might still argue that the *quality* of the evidence arrived at through intervention—and thus the confirmatory power of interventionist studies—is superior to that arrived at through non-interventionist means, such as observation, modeling, or natural experiment.

### Intervention and confirmation

Okasha (2011) defends a version of this view. According to him, experiments are epistemically superior to observational studies insofar as they are more likely to present strong evidence for universal generalizations of the form ∀x(Fx → Gx) than observational studies. Observational studies, he argues, involve merely “happening upon” some entity, *a*, that has both the properties of being F and G (i.e., happening upon some *a* for which “Fa & Ga” is true), while true experiments involve deliberate intervention to bring about Fa and to then test *a* to see whether “Ga” or “¬Ga” obtain. As a result, researchers in observational studies may learn that the conjunction “Fa & Ga” or “Fa & ¬Ga” is true, while in the interventionist study they learn whether the conditional “Fa → Ga” is true. Interventions thus guarantee to corroborate or disconfirm universal generalizations of the form, ∀x(Fx → Gx), by testing “Fa → Ga” while observational studies cannot. This is because when we bring about that some a is F, Fa becomes part of our background knowledge with the prior probability of 1. Since we already know that *a* has the property F when we set about to test for property G, we can say that our experiment was a genuine test of the generalization ∀x(Fx → Gx), as a negative result (¬Ga) would disconfirm both the conditional Fa → Ga and the generalization ∀x(Fx → Gx). At the same time, as Okasha writes:

“If one then learns that Ga and conditionalizes, the probability of the generalization must increase – for Ga is a logical consequence of the conjunction of Fa and ∀x(Fx→Gx), and Fa has prior probability of 1.”

If, however, we learn that “Fa” at the same time that we learn that “Ga,” then we are only permitted to test “(Fa and Ga),” since Ga is implied by the latter but not the former. For this reason, Okasha concludes that while interventionist studies are experiments proper, observational studies, including classic cases such as Sir Arthur Eddington’s 1919 expedition to test Einstein’s general relativity theory, are what he calls “rudimentary experiments” (Okasha, 2011). Rudimentary experiments share important features with experiment proper. Eddington, for instance, had to actively position himself and his instruments to ensure that he would receive the information needed to test relativity theory. This epistemic positioning, which Okasha dubs “organized observation,” is what permits Bayesian updating that transforms mere observation into a rudimentary experiment.

Okasha’s account thus has the resources to broaden the category of experiment to include instances in which researchers place themselves into appropriate epistemic positions without intervention. Insofar as he is only willing to grant such studies the status of “rudimentary” experiment, however, his rendering of experiment proper is overly narrow in several respects. First, many experiments—including experiments that involve interventions—are not aimed at testing laws or corroborating hypotheses, much less hypotheses in the form of universal generalizations. These include preparatory experiments, exploratory experiments in which the researcher does not test hypotheses at all, measurement experiments, and studies that are aimed at discovering entities rather than laws (Kästner, 2017; Waters, 2007).^12^ For example, although the results of the Michelson-Morley experiment were crucial for Einstein’s theory of general relativity (which did posit laws) the experiment itself was designed to test for the presence of a specific substance: viz., the aether (Shankland, 1964). Such experiments represent a substantial proportion of all experiments, and it would be odd if we could not learn from them, or could only learn relatively little, or if the information we gained were less reliable.

Second, casting such studies as Eddington’s expedition as mere *happenings upon* or as *rudimentary* mischaracterizes the labor and expertise that observational studies require—labor that Okasha himself acknowledges—thereby obscuring the epistemic contribution of expert observational knowledge. By first determining which sort of observation would corroborate a hypothesis, then identifying the precise conditions for observing the phenomenon (or its absence), and, finally, by taking the steps to bring the phenomena into view for an expert observer, the researcher conducting the observational study is actively and deliberately seeking out rather than passively chancing upon the phenomena of interest. For his part, Eddington together with Einstein dedicated significant effort to identifying the best means of providing empirical support for relativity theory (stellar parallax), the conditions that would permit him to observe it (a total lunar eclipse), and the best viewing location (one that had little to no cloud cover and was on land). His first planned expedition was cancelled due to a civil war and his second, successful, expedition required the coordination of many parties to transport him, his team, and their instruments to the island of Principe off the African coast. Once there, the observational study of stellar parallax required not only further coordination of multiple parties, but deep shared understanding of the instruments that expert observation and measurement required. Similarly, observational studies in medicine (e.g., of vaccine efficacy in real-world populations following placebo-controlled interventionist studies) are highly structured and meticulously planned. The preparation needed to carry out an observational experiment, in other words, requires no less careful planning, physical activity, and expert knowledge and know-how than do preparations for interventionist experiments.

Of course, Okasha’s primary insight is not about whether observational studies are active or passive or whether the data are obtained by happenstance or careful planning, but the comparative confirmatory power of observational and interventionist studies. Interventions have greater confirmatory power on his view because observational studies cannot first bring about that some entity, *a*, has property F before testing *a* for another property, G. As Okasha writes, “[the] important feature is that this involves learning that a given object has property G, where one did not know in advance that the object had property F” (Okasha, 2011). However, of those interventionist experiments that aim to test universal generalizations of the form ∀x(Fx → Gx), few begin by deliberately instantiating property F in the test entity (*a*). Instead, these studies identify an entity, *a*, that is antecedently known to possess F and then test it for property G. For example, testing the hypothesis that social intelligence (F) correlates with causal reasoning (G) does not involve first engineering an animal (a) to be socially intelligent (Fa). Rather, animals who are known from earlier studies to possess social intelligence (Fa) are then tested on causal reasoning tasks to determine whether they are also competent causal reasoners (Ga). In these cases, “Fa” is known antecedently but is not deliberately brought about through intervention; instead, it is discovered by independent experimental or observational means.

Put more generally, the universal generalization ∀x(Fx → Gx) is corroborated equally by the hypothesis “Fa → Ga” whether we engineer the antecedent, Fa, or whether we identify it in the wild. The simple Bayesian conditionalizing Okasha uses to make his case suggests that the prior probability assigned to “Fa → Ga” should not be affected by *how* we learn that Fa. If this is correct, then a study that does not intervene to bring about Fa can possess the same confirmatory power as one that engineers Fa. Thus, any observational study or natural experiment that first identifies Fa and then tests it for G will have the same confirmatory power with respect to universal generalizations of the form ∀x(Fx → Gx) as an interventionist study as long as the scientist first *discovers* Fa and only subsequently tests it for property, G. Many observational studies and natural experiments similarly locate the initial conditions and then observe the system unfold (in real time in the case of observations or retrospectively through forensic analysis in the case of natural experiments). If this representation of what is learned from such studies is correct, then many *interventionist* experiments would likewise only weakly corroborate universal generalizations.^13^ Furthermore, observational studies in medicine, such as the vaccine effectiveness studies mentioned above, may yield more reliable data than preliminary placebo-controlled studies if they (a) are more highly powered due to having a larger sample than the placebo-controlled studies; or (b) if they have superior ecological validity.

Finally, the case of sophisticated instrument-mediated imaging studies calls into question both the characterization of observational studies as passive *happenings upon* and the notion that they generally present poorer evidence than intervention. These include, e.g., the second-generation interferometers at the Laser Interferometer Gravitational-Wave Observatory (LIGO); the giant telescopes at the KECK observatory, which are outfitted with “adaptive optics” that permit fine-grained detection of astronomical phenomena by correcting for atmospheric occlusion in real time; and immense neutrino detectors such as TK2 and Icharus, which detect these elusive particles, which are so tiny that they very rarely interact with matter, with the aid of tremendous volumes of a medium (such as purified water or liquid argon) that increase the odds of a neutrino interaction combined with instruments that detect and record these interaction.^14^ The design, construction, and continuous re-calibration of these highly complex instruments requires immense intellectual capital involving collaboration among international teams of scientists and engineers. Studies conducted with the aid of these instruments have variously been characterized as observational and as experimental and have led to non-accidental discoveries such as the existence of super-massive black holes (Ghez et al., 2008). That these technologies can be used for precise structured observation suggests that the boundary between experiment and observation is porous (Perović, 2021; Boyd & Mathiessen, 2024), especially when observation is mediated by sophisticated detection and measurement technologies.^15^

Consider the case of LIGO. Thousands of physicists and engineers collaborated on the development and production of the instruments that comprise the two sprawling laser interferometers, each several kilometers in length and separated by 3,000 km. Because detecting gravitational waves requires great precision, the exquisitely sensitive detectors needed to be guarded against local interference from their surroundings, which required seeking out and building the devices in remote locations away from potential sources of human or environmental interference. The paired sites serve to corroborate the findings in any one instrument—a crucial role given the sensitivity of the instruments and consequent risk of false positives—serving as another means of control. The choice of site was critical for the success of the enterprise, as was the construction, calibration, and re-calibration or both interferometers. One might note here a parallel between the design and construction of LIGO and the plan and execution of Eddington’s expedition. The “initial run” of LIGO served as a prototype, which did not detect any gravitational waves but did permit the operators to refine the instruments. The “second run” was launched in 2014 with the first direct detection of gravitational waves taking place in 2015, when LIGO registered gravitational waves emitted by the collision of two massive black holes. The discovery of gravitational waves was predicted by, and thus provided significant corroboration for, Einstein’s General Relativity Theory—100 years after Einstein predicted the existence of gravitational waves.

Both the gravitational wave detection at LIGO and Eddington’s observation of stellar parallax share the same logical structure: (a) both were driven by a hypothesis that was derived directly from general relativity theory, and thus corroborated the theory; (b) neither Eddington nor the astrophysicists at LIGO brought about the antecedent, “Fa” (the eclipse and the collision of two massive black holes, respectively); and (c) both required a combination of precise instrumentation and observational expertise, which jointly permitted the researchers to identify and take epistemic advantage of the optimal location for viewing and measuring the phenomena of interest. Thus, both forms of study (i) corroborated a hypothesis about the law-like character of the physical universe despite (ii) involving no intervention in the system of study; and they were able to do so due to (iii) the researchers’ technical and observational expertise, combined with their expert knowledge about how best to bring the phenomena of interest into view.

### Bringing about versus bringing into view

A major difference between observational and interventional studies, then, seems to be that whereas interventions bring the phenomena *about*, observations bring them *into view*. Once the phenomena obtain, both interventionist and non-interventionist researchers observe, measure, and record. The observations and measurements themselves require years of training and practice, during which the researcher must learn not only how to calibrate and read the outputs of the instruments (in cases where observation is mediated by instruments) but also how to parse the visual or auditory scene, sometimes with the aid of other instruments. Even in observational studies that are not mediated by complex instruments, such as in ethological field studies of animal behaviors or anthropological studies of human populations, trained observational expertise is crucial for extracting meaningful information from apparently unstructured phenomena (Daston and Gallison 2007; Andrews, 2021). In these cases, it is the instruments together with observational expertise—and not intervention—that permit the accurate identification of phenomena.

One might object that although some instrument-mediated discoveries, such as the gravitational wave detection studies at LIGO, are epistemically on a par with experiments, this is only because they do, in fact, involve intervention insofar as they require the construction and calibration of sophisticated imaging technologies. However, while it is true that these instruments are designed by epistemic agents with the intention of bringing the phenomena into view, it is not clear that instrument construction satisfies Peschard’s definition of intervention. After all, the instruments neither bring the phenomena about nor effect a perturbation in the target system. Further, adopting this broader notion of intervention would force us to conclude that observational studies conducted with the aid of (e.g.) eyeglasses, might also count as an interventionist experiments.

## The practical value of intervention

Thus far, I have reconstructed and critiqued two arguments in defense of view that interventionist studies are (ceteris paribus) superior to non-interventionist studies. I have suggested that observational studies and natural experiments may be capable of identifying causal events and regularities and that the evidence delivered by structured observation may be on a par with the evidence delivered by interventionist studies. These arguments are unlikely to be exhaustive; my hope is that the foregoing discussions serve as a preliminary case against the received view of experiment.

If intervention is not necessarily superior to non-interventionist scientific studies, then what accounts for the durability of the intuition that intervention is a uniquely powerful tool in the scientific arsenal? Is it just a widely held false belief? Here, I propose the following heterodox answer: that the advantages of intervention are either practical rather than epistemic or not unique to classical experiment. These advantages are what we might call *convenience* and *access*. Intervention offers convenience insofar as it permits researchers to conduct experiments without the hassle of having to wait for the phenomena to come about on their own, which also allows researchers to attempt to repeat the investigation in an effort to replicate, and thus corroborate further, earlier results. In some cases, no amount of waiting or forensic analysis would bring the phenomena into view (e.g., as with the creation of new subatomic particles using the Superconducting Supercollider). In these cases, intervention may be needed to bring the phenomena about, which thus grants researchers special ‘access’ to otherwise inaccessible phenomena (see Keyser, 2021 for discussion of what he terms “intervention-based experimental production”). However, as I will argue below, convenience is practical rather than epistemic while access may be generated by instrument-mediated observation.

Consider two examples of convenience drawn from cognitive ethology and astrophysics, respectively. The ethologists Dorothy Cheney and Robert Seyfarth spent several years carefully observing the behaviors of a troupe of baboons in Botswana’s Okavanga Delta, which they chronicled in their popular book, *Baboon Metaphysics* (2009). Baboon societies are hierarchical and matrilineal and individual’s social status is partly a function of her kinship relationship to other females. Lower-ranked individuals tend to defer to higher-ranked individuals, and, where conflicts occur, lower-ranked baboons tend to defuse the situation by emitting yips of submission in response to barks of aggression by the higher-ranked individual. By observing the troupe’s social interactions over many years, Cheney and Seyfarth concluded that baboons understand and are able to keep track of changes to the hierarchy following births, deaths, successful uprisings, and migration into and out of the troupe. However, while they found their own observations convincing, they chose to conduct an experiment to further corroborate their hypothesis that the baboons were acting on social knowledge and to rule out the deflationary explanation that baboons were simply reacting to learned associations. They devised a series of playback experiments to test whether baboons would react with surprise to a violation of the social hierarchy, such as a higher ranked baboon letting out a submissive yip in response to an aggressive bark by a lower ranked baboon. According to the ‘expectation violation’ paradigm in psychology, we can infer that a subject has a belief if she reacts with surprise when that belief appears to have been violated. Surprise may be measured by looking time, since individuals who have expectations about some state of affairs should study the scene longer when the state of affairs does not obtain than when it does in order to understand how and why their predictions failed. In the experiment, Cheney and Seyfarth recorded calls from dominant and subordinate individuals, recombined them into the unusual sequence, and played this unusual sequence back to third party ‘bystander’ baboons when the subjects of the recording were out of view of the bystander. They found that bystanders looked longer in the direction of the recording when the sequence violated the hierarchy than when it did not. Thus, the playback experiments corroborated Cheney and Seyfarth’s observations that baboons understand social hierarchies.

Note that had nature happened to provide Cheney and Seyfarth with numerous instances of hierarchy-violations in the course of their observations, then the evidence from those instances would have had the same logical structure as the playback experiments. Because violations of the social hierarchy are rare but not impossible, they could in principle have had access to this evidence through expert observation alone. The intervention of the playback in the experiment was a great convenience, but it did not deliver stronger evidence than observation alone might have done with some luck, significant observational expertise, and more time.

Consider another example. The Japanese neutrino detector experiment, TK2, consists of a large neutrino detector and a neutrino generator located hundreds of miles apart. Rather than waiting for neutrinos to be generated by a galactic event, as other neutrino detectors do, TK2 allows researchers to control when emission (and, thus, detection) occurs, thus providing additional opportunities to study these elusive particles. These additional opportunities are a great convenience, accelerating the pace of research, but this convenience is logically independent from the strength or reliability of the evidence from TK2. Thus, the convenience of intervention offers a powerful advantage, but, insofar as the convenience does not affect confirmatory power or causal inference, we might say that this advantage is practical rather than epistemic.

Next, consider ‘access.’ Some configurations of nature may never present themselves to human beings given the limitations of our evolved perceptual apparatuses or the physical conditions of our corner of the universe. In such cases, interventions may permit scientists to bring the phenomena about (e.g., with the aid of particle colliders) that would never have come about naturally on Earth.^16^ However, intervention is not the only means of gaining special access to phenomena that is otherwise invisible to Earth-bound medium-sized creatures such as ourselves. Sophisticated detectors and imaging technologies, such as microscopes, telescopes, interferometers, MRIs, neutrino detectors, etc., also extend our evolved perceptual apparatus, giving us unique access to phenomena that would not have been observable—and, hence, knowable—without this technology.

In some cases, novel imaging technologies can make earlier interventionist strategies obsolete and may even improve the fidelity of the study. Consider, for example, the introduction of radioactive tracing to in vitro experiments in the molecular life sciences.^17^ An in vivo system, according to Roger Strand (1999) is “a biologically interesting, but experimentally inaccessible system” while “the corresponding in vitro system [is] a related, experimentally accessible, but biologically less interesting system,” where what counts as in vivo/in vitro depends on the “context of the particular research project” (Strand, 1999, 273–4). According to Hans-Jörg Rheinberger (2017), early 20th Century molecular biology aimed to cultivate a purified test tube environment that would leave the phenomenon as isolated as possible while preserving its native behavior. He writes:

“[a]round 1900, what was at stake was the fixation of the epistemic conditions under which it [was] possible for processes occurring within the organism to manifest outside the organism and thus to *become accessible* to analytic investigation. The enjeu is the creation of test tube environments in which ... biological entities are exposed to measurements, entities that are *otherwise hidden from the scientific gaze*, buried deep in the cell or the organism as a whole.” (p. Rheinberger, 2017, 280; emphasis added).

Note that Rheinberger frames the field’s early aim in terms of making visible the otherwise occluded. Similarly, Strand et al. (1996) describes in vitro effect studies as “address[ing] *inobservable* in vivo phenomena.” The isolation (extraction, purification) processes and their stabilization in an empirically tractable “test tube environment,” however, must be balanced against the need to preserve those factors that support the in-vivo behaviors under investigation. Further, it is not always possible to know in advance which features of the in vivo environment are necessary for the process to unfold as it does in the in vivo system (see Strand 1996 and 1999 for extended discussions).

Fortunately, the need to recreate the relevant features of the testing environment while simplifying it for the purposes of tractability lost some urgency with the advent of radioactive tracing. Rheinberger thus continues that “radioactive tracing in the test tube meant the possibility of going beyond the boundaries of chemical measurements that had previously relied on the availability of microgram mounts of substances in purified form—radioactive measurement could proceed in an essentially unpurified background” (Rheinberger, 2017, 287). By reducing their reliance on imperfect stabilization strategies, researchers could now begin testing molecular behavior in systems that more closely paralleled the native (in vivo) environments in which these processes normally unfold. Crucially for our purposes, this technological innovation meant that previously invisible processes could now be visualized with less intervention. In fact, Rheinberger goes as far as to liken radioactive tracing to electron microscopy—an imaging technology. He writes:

“After measuring devices had been developed that were sensitive enough to efficiently register the decay of tritium (3H) and radioactive carbon (14C), and after isotopes had become available en masse as a by-product of reactor technology (Creager, 2013), *the method of radioactive labeling functioned like some sort of biochemical electron microscope.”* (Rheinberger, 2017; emphasis added).

“Since then,” he concludes, “gene technology, itself a result of the development of molecular biology, is … choosing anew … *the intact cell* and *even the organism* as the space of its experimental intervention.” (288; emphasis added). In other words, Rheinberger seems to cast the field’s trajectory toward less interventionist imaging strategies as an overall epistemic good.

Technological advances such as these are especially important replacements for interventionist strategies that either threaten the integrity of the target system or that would be unethical to perform.^18^ Indeed, some phenomena cannot be studied except through non-interventionist means (e.g., measurements using microscopy), suggesting that non-interventionist studies, too, offer unique access to events that would be closed to interventionist studies. Thus, although the advantages of intervention are important, they may be either practical or, as in the case of access understood as making visible that which was theretofore invisible, not unique to intervention.^19^

My modest aim thus far has been to offer reasons to question the common, albeit often unstated, belief in the virtues of intervention in the context of scientific experiment. Much more work needs to be done to make a decisive case against what I have referred to as the received view of experiment. However, if the above arguments go through, then we should have less reason to believe that intervention accounts for the privileged epistemic status of experiment. Either experiment is not ceteris paribus superior to non-experimental studies, or a new definition of experiment is needed: one that does not lean so heavily on the concept of intervention.

One might wonder why redefining experiment is necessary. Why not simply reject the *epistemic superiority of experiment thesis* but retain the *experiment as intervention thesis*? Boyd (2023), for instance, advocates for decentering the role of experiment in philosophical analyses, writing that, “Rather than attending to the presence or absences of experiments to investigate the epistemology of science, we ought to instead attend to the target of the research and the processes that produce the empirical data.” I am sympathetic to this approach. However, the trouble with retaining the traditional account of experiment, as I see it, is that it may preserve the problematic incentive structures that grants unfair advantages to disciplines and research programs that are perceived as “more experimental.” Thus, in the next section, I sketch one provisional alternative proposal.^20^

## Experiment reimagined

If intervention neither defines nor explains the success of experiment, then, to adapt an expression from the cognitive sciences, what might be “the mark of the experimental” (Allen, 2017)? I believe that an answer may lie with Okasha’s insight that it is the epistemic positioning of the researcher that matters. But what does it mean to epistemically position oneself in the right way? In what follows, I sketch a *preliminary* account of experiment in terms of what I call “deliberate positioning,” which centers not on intervention, but on control.

The introduction to the first major edited volume on the history and philosophy of controlling strategies and control concepts opens with the following bold proclamation: “Control is the hallmark of scientific experimentation” (Schickore, 2024, 1). Yet control has been comparatively undertheorized in the philosophy of experiment, with the generative, phenomena-producing, power of intervention taking center stage instead (Arnet, 2024). Schickore (2024) identifies two senses of control: (1) the *narrow sense* of control “as a comparative trial” (e.g., the familiar segregation into ‘control’ versus ‘treatment’ groups in RCTs, etc.) and (2) control in the broad sense of “‘managing,’ ‘restraining,’ or ‘keeping everything stable except the target system to be intervened upon.” She situates her account of control in the context of interventionist experiment, so the reference to intervention in the broad definition is not surprising. Neither, however, is it strictly necessary. For instance, Arnet (2024) glosses control in the broad sense as “an intended purificatory process in which scientists attempt to stabilize intervening variables to *expose the contours and nature* of a phenomenon *or* an intervention” (Arnet, 2024, 284; emphasis added). Both Eddington and Snow were able to achieve control in this latter sense by deliberately positioning themselves—in physical space and through forensic analysis, respectively—with respect to the subject they wished to investigate in order to isolate the target phenomena. In both cases, this also required expertise and know-how. In light of these considerations, I would like to propose the following definition sketch:

- **Experiment is** the deliberate positioning of a researcher with respect to some aspect of the physical universe and relative to their specific epistemic goal, which can actually or effectively isolate the phenomenon of interest in order to control for confounds, is in principle repeatable, and has the potential to generate new discoveries about the target system.

Let us unpack this. I understand deliberate positioning as any of a number of strategies—from manipulation of instruments, to crafting an ethogram of animal behaviors, to preparation of a sample, to statistical retrofitting, and more—that give the researcher the ability to isolate the phenomenon of interest in order to control for possible confounds. It can look like Eddington’s observational experiment, like instrument-mediated measurement experiments such as those that led to the first discovery of the supermassive black hole at the center of our galaxy, like modeling practices ranging from genetic sequencing work based on model organisms to perturbations of scale models of traffic flow, and beyond. The epistemic goal may be exploratory or hypothesis-driven; it may seek causal explanation, or it may aim to map constitutive elements of a system; it may be accomplished within a laboratory, field, or hybrid setting; and its realization may or may not require or permit intervention, depending upon the nature of the target system and the epistemic goal.

Isolation refers to the separation of the target phenomenon from potential confounding factors, which I, following Currie and Levy (2019), take to be necessary for control. They write that “an object is subjected to control when isolated from its natural environment and intervened upon in a replicable way” (2019).^21^ However, while Currie and Levy take control to require not only isolation, but also in-principle replicability and intervention,^22^ this account does not. In this respect, the deliberate positioning definition-sketch is consonant with the majority view of control in the broad sense, which permits, but does not require replication or intervention.^23^ Isolation might be achieved with the aid of intervention by taking a representative sample of the target population into a laboratory and preparing it for study by purifying it until only what Currie and Levy (2019) call “focal properties”—i.e., those about which we wish to learn—remain. However, it may also be achieved through expert observation, with or without the aid of imaging instruments such as adaptive optics, through forensic analysis combined with appropriate statistical methods (see Morgan, 2013; Spirtes, 2010), or with the aid of simulations and models.

Furthermore, strategies that isolate a target phenomenon may be replicable even if they are not interventionist. For example, one might examine multiple “runs” of the observational study in cases where natural experiments have been “naturally replicated,” such as multiple instances of evolutionary convergence on a trait. Instrument-mediated observations such as those conducted at LIGO and KECK observatories offer some of the clearest examples of highly controlled observational experiments that isolate the target phenomenon without intervention and can do so multiple times, permitting replication.^24^ These studies in particular press us to reexamine the nature of isolation, control, and experimentality.^25^

Finally, experiment must be capable of generating new discoveries or corroborating previous ones to ensure that such passive learning strategies as attending a lecture, reading a book, or searching the internet are excluded from the definition of experiment. At the same time, the deliberate positioning view is broad enough to include such practices as archival research, as this research can generate new knowledge about the world. Some may regard any definition of experiment that accommodates research in humanities fields such as history as problematically capacious. However, whether some humanities research counts as experimental depends on whether their methods permit isolation and control. Thus, the deliberate positioning account can in principle exclude all humanities research; it simply does not presuppose that this research ought to be excluded. Even setting the humanities aside, however, one might worry that a liberal definition of experiment would paper over important distinctions among experimental, observational, and modeling strategies. However, I see no barrier to retaining these distinctions within the broader class of experiment, either as sub-types of experiment (e.g., as “observational experiment,” “modeling experiment,” etc.) or as independent strategies within experimental practices. Indeed, the latter may be preferable in light of the fact that a given experiment typically involve multiple strategies at once (Currie & Levy, 2019).

A full defense of the deliberate positioning account is beyond the scope of the present paper. It may be that *experimentation* is a category much like *life* is in biology (Cleland & Chyba, 2018; Machery, 2012; but see Ruiz-Mirazo et al., 2004; see Mariscal et al., 2019 for a review) or *cognition* is in cognitive science (Bräuer et al., 2020; Allen, 2017; but see Keijzer, 2021): at once discipline-defining and resistant to definition. Perhaps experiments bear a family resemblance to one another; or, perhaps many mutually irreducible definitions of experiment are possible. I will not take a stand at this time. However, if something like a definition is desirable—perhaps because such definitions serve the heuristic purpose of identifying epistemically superior forms of scientific study—then it should at minimum home in on the unique features of experimentality. By emphasizing control over intervention, the deliberate positioning account preserves the intuition that experiment is superior to other forms of study but redefines experiment to include other controlled scientific investigations. Furthermore, nothing in the deliberate positioning account precludes analyses of the unique virtues and limitations of interventionist experiments (see §4), observational studies, natural experiments, or model-based experiments. It therefore preserves crucial distinctions, eliminating only the presumption of epistemic inferiority of non-interventionist studies.

Most importantly, both the deliberate positioning account and deflationary approaches may level the playing field in terms of resource allocation between scientific disciplines that are currently regarded as experimental and those that are viewed as non-experimental. Although I cannot hope to fully defend this suggestion in space remaining, it seems plausible that where perceptions of rigor are tied to perceptions of experimentality and where experimentality is glossed in interventionist terms, resources (grant funding, fellowships, successful peer review, etc.) would be expected to flow to individuals in disciplines or research programs that are perceived as more experimental or that adopt more interventionist strategies. Shifting the structure of resource allocation could, in turn, shift the incentive structures that determine which disciplines draw the most funding and talent and which methodologies come to be adopted within a given discipline. Reimagining the concept of “experiment” may have two additional and related advantages: First, it may increase access to resources (e.g., funding and mentorship opportunities, academic posts, publications) for putatively non-experimental fields, making them more attractive to new talent and better provisioning the kinds of research typically undertaken in those fields (see Perovic 2021 for additional discussion). Second, and relatedly, it may remove incentives to adopt suboptimal research methods in contexts where intervention is inappropriate. In short, more inclusive accounts of experimentality may incentivize the adoption of more field-appropriate strategies as well as a more equitable distribution of critical but limited resources.

## Summary and conclusion

Let us take stock one final time. I began this paper by reconstructing and rejecting two arguments for the thesis that interventionist scientific investigations are epistemically superior to non-interventionist investigations. The first argument held that causal relationships are central to scientific explanation and that uncovering these requires intervention. I argued that (i) non-causal relationships are also significant and that uncovering these may require non-interventionist strategies; and (ii) that natural experiments and observational studies can produce knowledge about causal relationships. The second argument held that intervention secures better evidence than studies that employ non-interventionist strategies. I explored and critiqued one specific version of this argument by Okasha (2011) and argued that classical observational studies that are similarly hypothesis-driven may have the same logical structure as interventionist experiments. Instrument-mediated “observational” studies in particular press us to reconsider how control may be achieved by means other than intervention. Building on Kästner (2017), I suggested that whereas interventions may bring phenomena *about*, non-interventionist strategies, including but not limited to observational studies, *bring them into view*. Next, I considered the possibility that if intervention carries any unique advantages, these may be practical rather than epistemic and consist in what I called *convenience* (e.g., not needing to wait for rare events to occur on their own) and *access* to phenomena that would not naturally occur or that could not be perceived by limited Earth-bound beings such as ourselves. At the same time, non-interventionist strategies, such as instrument-mediated observations, also permit access to phenomena that would be undetectable and thus impossible to measure without them, blurring even this line between the merits of interventionist and non-interventionist science.

While these arguments do not establish epistemic parity between interventionist and non-interventionist strategies, they put pressure on the received view of experiment. This, in turn, invites us to ask how experiment might be reimagined in non-interventionist terms. The “deliberate positioning” account with which I closed this essay charts one of several possible paths in this direction, giving proper credit to investigations in the more historical, observational, and model-based fields and research practices and foregrounding the value of reorienting philosophy of experimentation away from intervention and toward theories of control. Whether or not it ultimately succeeds, I hope to have motivated the idea that a revised account is not only possible, but that it may be desirable on both epistemic and practical grounds.

## Data Availability

The article has no supporting data, and thus no plan for making data available is required.

## Footnotes

1 Boyd and Mathiessenn (2024) and Currie and Levy (2019) are the rare exceptions. Boyd and Mathiessenn train their criticisms on the experiment/observation dichotomy, urging philosophers to focus instead on the manner in which data is generated. Currie and Levy defend only the claim that “experiment are privileged means of confirmation,” thus explicitly restricting their analysis to experiments that seek confirmation—i.e., hypothesis-driven experiments. They do not endorse the broader claim that experiments simpliciter are ceteris paribus superior to other kinds of studies.

2 For instance, Zweir writes: “I wish to include anything that we might naturally call an “experiment”—that is, a scientific study in which the investigator deliberately sets up and/or intervenes on a system for the purpose of studying it” (Zweir 2013, 661).

3 For example, Woodward’s (1997) “mutual manipulability” account of causation defines a causal relationship between two elements of a system in terms of *hypothetical interventions*. Such counterfactual accounts require neither actual manipulation nor human agency.

4 Currie and Levy (2019) refer to representative samples of a target system as “specimens” and suggest that the representativeness of a specimen is secured by the fairness of the methods used in procuring and preparing the sample.

5 The details of this argument will vary with the account of causation and explanation, but the general form of the argument is enough for the present purposes.

6 While Snow’s methods presage contemporary controlling strategies, Snow himself did not use the contemporary term, “control.” As Schickore (2024) writes, “controlling practices and strategies predate the appearance of the term ‘control’ in the scientific literature.”.

7 The full story is appreciably more complex. For instance, Snow’s hypothesis made epidemiological assumptions about disease transmission (viz., that illnesses could be transmitted via water) that competing hypotheses, such as the leading miasma theory could not easily assimilate (Tulodziecki 2011). See Tudolziecki (2011) for a detailed analysis of Snow’s explanatory success, which she attributes to the superior explanatory power of Snow’s theory relative to the other leading theory of cholera at the time. Tulodziecki is especially interested in how Snow was able to determine the causes of the outbreak without knowing the mechanism by which cholera was spread (viz. that it was the infectious and transmissible bacterium, *vibrio cholerae*).

8 Indeed, the notion that intervention requires the intentional production of effects by a human agent seems to gloss over the complexities involved in measurement even within traditional interventionist experimental contexts. As Keyser (2017) writes, “At a certain point, a scientist’s skillful manipulation of an intervention system stabilizes both in physical conditions and representation… That is, I see the process of production in terms of controlling conditions to yield *repeatable, reproducible, and reliable* intervention systems—where the purposes of the experiment dictate which of these parameters is the center of attention” (p. 20–21). Keyser (2019, 2021) adds that in the production of artefacts, intervention systems, and effects there are deeper entanglements and forms of ‘co-stabilization’ between nature, measurement process/system, and experimenter.

9 Even if it did not reduce intervention to mere causal influence, it would, at a minimum, require abandoning the “thick” version of intervention that underpins the received view.

10 Morgan suggests calling the former *nature’s* or *society’s* experiments and reserving the concept of *natural experiment* for the latter case.

11 Cleland attributes the view that historical sciences are incapable of causal explanation to the inaccurate perception that explanations in historical sciences take a primarily narrative form and that narratives are inherently subjective.

12 Because Currie and Levy (2019) restrict their analysis to the superior confirmatory power of interventionist studies, their analysis faces a similar limitation.

13 One might object that the cases I have described do not genuinely test “Fa → Ga” because the entity already possesses both properties F and G. Thus, on this objection, the evidence that these studies deliver is better represented as “Fa & Ga” even if the researcher learns that Ga is true only after learning that Fa is true and even if they learn that Ga is true by conducting interventionist studies. If this objection is right, then such studies may provide weaker corroboration of ∀x(Fx → Gx) than studies that intervene to instantiate F in some *a* that is known to possess neither F nor G prior to the intervention and then test it for G. However, this objection misses the Bayesian point, since what matters is not the state of the system, but the researcher’s beliefs about that system.

14 Because neutrinos are so small, they can travel through atoms as though through vacuum, rarely making contact with other subatomic particles. To increase the odds of capturing a neutrino interaction, the volume of the medium in the detector must also be large: the greater the volume, the greater the chances of capturing a neutrino. However, the interaction is not detectable by means of unaided human vision, requiring technologies that measure, interpret, and visualize.

15 Boyd (2023) argues that, contrary to common philosophical wisdom, astrophysics is a partly experimental science insofar as it utilizes laboratory-based interventions on terrestrial phenomena that share critical properties with the astral phenomena of interest. She rejects the observation/experiment dichotomy, writing, “Instead of attending to the distinction between ‘observation’ and ‘experiment’ (or ‘observational science’ and ‘experimental science’) in our investigation of the epistemology of astrophysics, we should attend to the production of empirical data” (ibid).

16 Notice that if they could come about without human intervention but are simply unlikely to, then this form of access reduces to convenience.

17 I am indebted to an anonymous referee for directing me to this literature.

18 For example, Strand et al. (1996) present in vitro studies as those in which (inter alia): “it is methodologically impossible, inconvenient or unethical to study the phenomenon of interest” in the in vivo system.

19 An anonymous referee queries whether in vitro studies might involve a new kind of *access*: access as control. I have argued that access of this sort is simply another way to bring a phenomenon into view, but I suspect that a more complete answer must attend to the queer ontology of in vitro systems, which in some cases appear to function both as representative samples and as representational models of the target phenomenon. Under at least some circumstances, the preparatory process (isolation by means of extraction, purification, and the stabilization of the testing environment) may transform the sample into an analogue of the target (in vivo) system, then the isolation process transforms the subject system into a *representation* of the target system. Should this occur, we may say that isolation converts the sample into something functionally similar to a model system. This reading is suggested, for instance, in how Strand et al. (1996) describe a class of in vitro studies: in vitro effect studies, writing that “[i]n vitro effect studies address inobservable in vivo phenomena through experiments on *analogous* in vitro phenomena” (emphasis added). Even more to the point, Rheinberger (2015), writes: “Biochemical models of partial reactions of biological processes can be seen as a boundary case [between models proper and what he calls “preparations”]. On the one hand, their medium is the test tube, not the organism, on the other hand they rely on materials derived from organisms” (Rheinberger 2015, 325). And, if this is the case, then the interventions involved would be conducted not on the system of interest but on a *representation* of that system—much as with models. Under these circumstances, the epistemic benefits of the isolation process seem to accrue not to classical interventionist experiment, but to (something like) model-based studie.

20 An anonymous referee queries whether this strategy is likely to work, suggesting that the meaning of “experiment” may be too fixed in the minds of those who need to be persuaded. I am sympathetic to this concern, but would suggest that the *value* implicitly attached to experiment may be even more recalcitrant than its meaning. Further, empirically informed, research is needed to resolve this issue.

21 Crucially, the ‘object’ must be what they call a ‘specimen’—i.e., a representative sample or “a typical instance of the group.” This ‘typicality’ is cashed out in terms of similarity between the focal point of the object and the target.

22 Desjardins et al. (2023) argue that control as Currie and Levy understand it—viz., as requiring intervention—may impede scientific progress in certain historical sciences, writing that “emphasizing control in the wrong circumstances can mislead the investigation, or even destroy the intended object of investigation.” Were Desjardins et al. take issue with control, I take issue with weaving intervention into the concept of control. Indeed, their real target may be not control per se, but control that requires intervention. In other words, this is control in E.G. Boring’s second sense of control: viz., control as targeted manipulation (Boring, 1954).

23 I should note that others do include replication as a kind of control. For instance, writing about controlling strategies in the competing research programs of Carl von Heß and Karl von Frisch into color vision in fishes, Christoph Hoffman identifies control with experimental replication and other testing strategies: “Heß’s and Frisch’s experiments became the subject of control in many ways. They rarely used the term “control,” however (German: *Kontrolle*, *kontrollieren*), either alone or in combination with other expressions. Instead, they usually used related expressions ranging from *verifying*, *checking*, *testing*, and *copying* experiments, to *repeating* experiments or trying them once again. This terminology notwithstanding, there are many hints in their reports about procedures that, from an analytical point of view, are to be understood as control activities.” (Hoffman 2024, 294). These controlling activities are then further complicated, Hoffman suggests, when the research subject has agency of its own.

24 Indeed, we might make sense of the calibration and re-calibration processes in advance of instrument-guided experiments as an example Schickore’s (2019) “control-experiments,” the aim of which is to probe for possible confounders. With respect to complex instruments, control-experiments will often include “debugging” of the instruments themselves, which is a necessarily exploratory process insofar as the process is designed to ferret out unknown problems. Once a “confounder repertoire” is established, further calibration and re-calibration will aim at cancelling out or avoiding these confounders—in other words, calibration at this step becomes “control” in Arnet’s sense of “purifying processes.” Furthermore, complex instrumentation requires a large team of scientist and engineers, in part to attain greater inter-observer reliability and in part due to the complexity and scope of the instrumentation outstripping the expertise and capability of any single individual. As Schickore (2023) writes, even though sophisticated instruments “often make it easier to keep an experimental situation stable and to track interesting changes … closer analysis of actual episodes shows that advancements in instrumentation … do not guarantee improved control. In fact, obtaining control often becomes more difficult, not least because researchers must learn the instruments’ proper functioning” (Schickore 2024, p. 6). I am indebted to an anonymous referee for the question about the contributions of calibration and re-calibration in successful experiments.

25 Arnet (2024) suggests that control-oriented philosophies of scientific practice identify a feature of scientific practice that traditional approaches, which emphasize the *productive* power of interventionist experiment, tend to ignore. As he writes, “this [broad/historical] sense of control … emphasizes not the creation of laboratory phenomena [per Hacking] but rather the elimination of interfering nature…. What is important to scientists working in these ways is precisely that the phenomenon is not “created,” but rather that it has a fidelity to nature that can be achieved only in the structured context of the experiment” (Arnet 2024, 269–70). I take myself to be engaged in a similar project insofar as I endorse moving away from intervention-focused approaches to scientific practice and toward those that places a greater emphasis on control.
